# Trends in surgical treatment of femoral neck fractures in the elderly

**DOI:** 10.1590/S1679-45082018AO4351

**Published:** 2018-09-09

**Authors:** Eva Jolanda Irene Lehtonen, Robert Davis Stibolt, Walter Smith, Bradley Wills, Martim Correia Pinto, Gerald McGwin, Ashish Shah, Alexandre Leme Godoy-Santos, Sameer Naranje

**Affiliations:** 1 University of Alabama, Birmingham, AL, United States.; 2 Hospital Israelita Albert Einstein, São Paulo, SP, Brazil.

**Keywords:** Aged, Hip fractures/surgery, Femoral neck fractures/surgery, Surgical procedures, operative/trends, Idoso, Fraturas do quadril/cirurgia, Fraturas do colo femoral/cirurgia, Procedimentos cirúrgicos operatórios/tendências

## Abstract

**Objective:**

To analyze recent demographic and medical billing trends in treatment of femoral neck fracture of American elderly patients.

**Methods:**

The American College of Surgeons National Surgical Quality Improvement Program database was analyzed from 2006 to 2015, for patients aged 65 years and older, using the Current Procedural Terminology codes 27130, 27125, 27235, and 27236. Patient demographics, postoperative complications, and frequency of codes were compared and analyzed over time. Our sample had 17,122 elderly patients, in that, 70% were female, mean age of 80.1 years (standard deviation±6.6 years).

**Results:**

The number of cases increased, but age, gender, body mass index, rates of diabetes and smoking did not change over time. Open reduction internal fixation was the most commonly billed code, with 9,169 patients (53.6%), followed by hemiarthroplasty with 5,861 (34.2%) patients. Combined estimated probability of morbidity was 9.8% (standard deviation±5.2%), and did not change significantly over time. Postoperative complication rates were similar between treatments.

**Conclusion:**

Demographics and morbidity rates in femoral neck fractures of elderly patients did not change significantly from 2006 to 2015. Open reduction internal fixation was the most common treatment followed by hemiarthroplasty.

## RESUMO

**Objetivo:**

Analisar tendências recentes demográficas e de faturamento médico no tratamento de fraturas do colo do fêmur em idosos americanos.

**Métodos:**

O banco de dados *National Surgical Quality Improvement Program*, do *American College of Surgeons*, foi analisado de 2006 a 2015, para pacientes com idade igual ou superior a 65 anos, usando os códigos de *Current Procedural Terminology* 27130, 27125, 27235 e 27236. Dados demográficos dos pacientes, complicações pós-operatórias e frequência de códigos foram comparados e analisados ao longo do tempo. A amostra teve 17.122 pacientes geriátricos, sendo 70% do sexo feminino, com média de idade de 80,1 anos (desvio padrão±6,6 anos).

**Resultados:**

O número de casos aumentou no período, mas idade, gênero, índice de massa corporal e taxas de diabetes e tabagismo não mudaram ao longo do tempo. A fixação interna de redução aberta foi o código faturado mais comum, com 9.169 pacientes (53,6%), seguido por artroplastia parcial do quadril, com 5.861 (34,2%) pacientes. A probabilidade estimada combinada de morbidade foi de 9,8% (desvio padrão±5,2%) e não mudou significativamente ao longo do tempo. As taxas de complicações pós-operatórias foram semelhantes entre os tratamentos.

**Conclusão:**

Os dados demográficos e as taxas de morbidade relacionadas às fraturas geriátricas do colo do fêmur não apresentaram mudança significativa entre 2006 e 2015. A redução aberta e a fixação interna foram as opções de tratamento mais comuns, seguidas da artroplastia parcial do quadril.

## INTRODUCTION

Hip fractures are among the most common injuries seen by orthopedic surgeons and are especially prevalent in the geriatric population. In 2014, there were over 320,000 hip fractures that presented to the emergency department in the United States with the majority occurring in women age 65 or older.^(^ ^1^ ^)^ This number has increased compared to the previous 5 years. Over one-third of adults age 65 or older suffer a fall each year.^(^ ^2^ ^)^ Increased numbers of falls in combination with increased susceptibility for fracture from age-associated decreases in bone mineral density, as well as the presence of other co-existing comorbidities makes the geriatric population particularly susceptible to fracture. Hip fractures decrease patient independence, mobility, and have been associated with increased risk of mortality.^(^ ^3^ ^)^ As the population continues to age the absolute number of falls, and subsequently the number of fractures, are set to increase.

There are a number of treatment options for fractures of the femoral neck. Typically, these fractures are classified as either non-displaced or displaced. Non-displaced fractures can be treated with internal screw fixation, although several studies have demonstrated internal fixation as a less optimal treatment, particularly in the elderly population.^(^ ^4^ ^,^ ^5^ ^)^ In very elderly or chronically ill patients, surgeons tend to proceed with a hemiarthroplasty (HA). Total hip arthroplasty (THA) has historically been preserved for younger, active patients that have a history of hip osteoarthritis. There have been many studies, however, that have shown function after THA to be superior to HA.^(^ ^6^ ^-^ ^14^ ^)^

In 2008, Jain et al. reviewed inpatient data from 1990 to 2001 and found that overall, regardless of age, there was a decrease in the use of THA for the treatment of femoral neck fractures.^(^ ^15^ ^)^ In contrast, Miller et al. looked specifically at the Medicare population from 1991 to 2008 and found that the percentage of hip fractures treated nonoperatively, with internal fixation, HA, or THA remained fairly constant over time.^(^ ^16^ ^)^ These studies utilized ICD-9 and ICD-10 coding, however, medical billers, resident case logs, and certain databases, like the American College of Surgeons National Surgical Quality Improvement Program (ACS NSQIP^®^) database rely on Current Procedural Codes (CPT) instead.

## OBJECTIVE

To analyze recent demographic and surgical coding trends in the management of femoral neck fractures for patients aged 65 or older using the Current Procedural Codes system.

## METHODS

### Data source

We performed a retrospective analysis of prospectively collected data using the ACS NSQIP^®^ database. NSQIP^®^ is a surgical outcomes database that maintains preoperative through 30-day postoperative data on randomly assigned patients from more than 400 academic and private community hospitals. NSQIP^®^ contains de-identified patient information, and no characteristics allowing for personal identification of patients are included in the data set. Therefore, this study was exempt from review by the Institutional Review Board.

Our study cohort included all patients aged 65 years old and over, who underwent surgical repair of femoral neck fracture from 2006 to 2015. The patients queried in our study were identified by CPT 27130, 27125, 27235, and 27236. These codes correspond to total hip replacement, partial hip replacement, placement of cannulated screws for femoral neck fracture, and open treatment of femoral fracture with either internal fixation or prosthetic placement, respectively. Patients with operative times less than 25 minutes or greater than 300 minutes were excluded, to avoid outlying data.

A total of 17,122 geriatric patients were included in our sample. Overall, our sample was 70% female with an average age at surgery of 80.1 years (standard deviation - SD, 6.6 years).

The primary outcomes of interest were the trends in: type of procedure performed between 2006 to 2015; pre-existing comorbidities (*e.g*. diabetes, smoking status, chronic steroid use) across the four CPT codes; and post-operative complications including deep vein thrombosis (DVT), cerebrovascular accident (CVA), surgical site infections, and pneumonias.

### Statistical analysis

All statistical analysis was performed using SAS 9.4 (Cary, NC). Trends in the demographic variables and frequency of THA, HA, and cannulated screw fixation, and were analyzed and plotted using a stacked column graph.

## RESULTS

Patient demographics were similar between surgical techniques despite differing group sizes. Combined estimated probability of morbidity of all patients was 9.8% (SD 5.2%).

**Figure 1:**
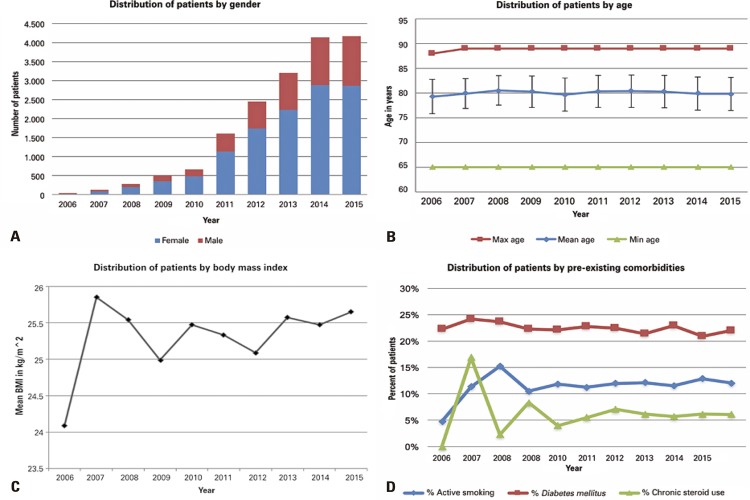
Distribution of patients by gender, age, body mass index, pre-existing comorbidities

Summary statistics of surgical demographics by procedure and trends in demographics over time are provided in table 1 and figure 1, respectively.

**Table 1:** Statistics of surgical and demographics by procedures

Variables	Mean±SD or frequency (proportion)				

	Overall	THA (27,130)	HA (27,125)	Cannulation (27,235)	ORIF/Pros (27,236)
Number of patients, n (%)	17,122	1,695 (9.9)	5,861 (34.2)	397 (2.3)	9,169 (53.6)
Age at surgery	80.1±6.6	76.2±7.1	80.7±6.3	79.6±6.9	80.4±6.5
Male, n (%)	5,131* (30.0)	507 (29.9)	1,792* (30.6)	100* (25.3)	2,732* (29.8)
Female, n (%)	11,986* (70.0)	1,188 (70.1)	4,068* (69.4)	295* (74.7)	6,435* (70.2)
BMI	24.9^+^	25.6*	24.6*	24.7*	24.9*
Height, in	64.9*±4.1	65.4*±4.1	64.9*±4.1	64.6*±4.3	64.8*±4.1
Weight, lbs	149.0*±37.1	155.8*±37.6	147.5*±36.5	146.7*±42.8	148.7*±37.0
Current smoker within 1 year, n (%)	1,838 (10.7)	200 (11.8)	645 (11.0)	48 (12.1)	945 (10.3)
*Diabetes mellitus*, n (%)	3,090 (18.0)	256 (15.1)	1,092 (18.6)	72 (18.1)	1,670 (18.2)
Chronic steroid use, n (%)	981 (5.7)	103 (6.1)	342 (5.8)	18 (4.5)	518 (5.6)
Preoperative hematocrit	36.3*±4.8	36.9*±4.7	36.2*±4.8	36.7*±4.9	36.3*±4.9
Operative time^+^, minute	75.9*±41.0	95.2±49.5	76.5*±41.7	34.8±19.8	73.7*±37.6
Estimated probability of morbidity (%)	9.8*±0.052	4.7*±0.025	10.6*±0.052	40.3*±0.0^†^	10.1*±0.051

* Missing data values, average or percent of recorded values given; ^†^ standard deviation not available due to lack of sufficient data. SD: standard deviation; THA: total hip arthroplasty; HA: hemiarthroplasty; ORIF/pros: open reduction internal fixation/prosthetic; BMI: body mass index.

### Surgical trends

**Figure 2:**
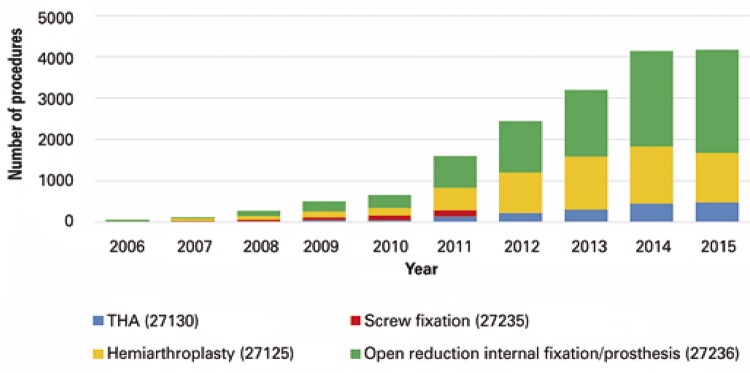
Distribution of procedures performed

Open reduction internal fixation/prosthetic placement (CPT 27236) was the most commonly performed procedure, with 9,169 patients (53.6%) from 2006 to 2015. Hemiarthroplasty (CPT 27125) was the second most common method of fixation, with 5,861 (34.2%) patients. Only 397 (2.3%) patients underwent internal fixation with cannulated screws, and there were no records of this procedure after 2011. Trends in procedure frequency over time are portrayed in figure 2.

THA: total hip arthroplasty.

### Postoperative complications

Many complications were not observed in any patient, or there were missing data from 2006 to 2010. Mean and standard deviation for the estimated probability of morbidity could not be calculated prior to 2011. Complication rates were similar between 2011 and 2015. Trends in the postoperative complications per year are shown in table 2.

**Table 2:** Summarized statistics of postoperative complications

Year	2006	2007	2008	2009	2010	2011	2012	2013	2014	2015
Total number of patients	22	118	272	494	662	1,603	2,444	3,201	4,136	4,170
Medical complications										
Readmission*	0	0	0	0	1 (10.00)	147 (9.17)	225 (9.21)	276 (8.62)	349 (8.50)	384 (9.21)
Pneumonia	2 (9.09)	5 (4.24)	13 (4.78)	14 (2.83)	18 (2.72)	60 (3.74)	71 (2.91)	144 (4.50)	174 (4.21)	178 (4.27)
CVA	0	0	1 (0.37)	0	5 (0.76)	10 (0.62)	10 (0.41)	31 (0.97)	34 (0.82)	22 (0.53)
Bleeding transfusions	0	0	2 (0.74)	1 (0.20)	124 (18.73)	421 (26.26)	651 (26.64)	734 (22.93)	770 (18.62)	637 (15.28)
Surgical complications										
Return to operating room*	0	6 (5.08)	3 (1.1)	16 (3.24)	22 (3.32)	54 (3.38)	93 (3.81)	95 (2.97)	141 (3.41)	163 (3.91)
Superficial incisional surgical site infection	0	0	4 (1.47)	7 (1.42)	6 (0.91)	15 (0.94)	22 (0.9)	24 (0.75)	28 (0.68)	28 (0.67)
Deep incisional surgical site infection	0	3 (2.54)	0	4 (0.81)	6 (0.91)	12 (0.75)	10 (0.41)	13 (0.41)	21 (0.51)	20 (0.48)
Organ/space surgical site infection	0	0	0	0	2 (0.3)	5 (0.31)	10 (0.41)	7 (0.22)	7 (0.17)	16 (0.38)
Wound disruption	0	0	0	0	1 (0.15)	2 (0.12)	4 (0.16)	2 (0.06)	7 (0.17)	6 (0.14)
DVT/thrombophlebitis	0	3 (2.54)	2 (0.74)	12 (2.43)	4 (0.60)	10 (0.62)	23 (0.94)	24 (0.75)	42 (1.02)	39 (0.94)
Pulmonary embolism	0	3 (2.54)	3 (1.1)	5 (1.01)	1 (0.15)	7 (0.44)	19 (0.78)	22 (0.69)	40 (0.97)	30 (0.72)
Estimated probability of morbidity	N/A	N/A	0.4032	N/A	0.1092	0.1339 (0.0810)	0.1155 (0.0577)	0.0904 (0.0491)	0.0900 (0.0484)	0.1008 (0.0521)

Results expressed as n, n (%), or median (SD). * Missing data points, percentage of available data. CVA: cerebrovascular accident; OR: operating room; SSI: surgical site infection; DVT: deep vein thrombosis; SD: standard deviation; N/A: not applicable.

Postoperative complication rates were similar for readmission, CVA, surgical site infections, wound dehiscence, DVT or thrombophlebitis, or pulmonary embolism (PE), regardless from the surgical technique. Patients who underwent cannulated screw fixation had the lowest complication rates for all complications evaluated. Patients who underwent THA had the highest rates of postoperative blood transfusions (23.42%), return to the operating room (4.42%), deep incisional surgical site infection (0.53%), organ space surgical site infection (0.29%), and wound disruption (0.18%). HA patients had higher rates of readmission (9.20%), postoperative pneumonia (4.74%), superficial surgical site infection (0.89%), DVT (1.04%) and PE (0.83%) when compared to patients treated with other techniques. Postoperative complication rates by procedure type are shown in table 3.

**Table 3:** Summary statistics of postoperative complications by procedure type

Complication	Overall	ORIF/Pros*	THA*	HA*	Cannulation*
Total number of patients	17,122	9,169	1,695	5,861	397
Medical complications					
Readmission^†^	1,235 (8.86)	673 (8.78)	116 (8.10)	446 (9.20)	-
Pneumonia	679 (3.97)	356 (3.88)	39 (2.30)	278 (4.74)	6 (1.51)
CVA	113 (0.66)	66 (0.72)	8 (0.47)	39 (0.67)	0
Bleeding transfusions	3,340 (19.51)	1,721 (18.77)	397 (23.42)	1,203 (20.53)	19 (4.79)
Surgical complications					
Superficial incisional surgical site infection	593 (3.46)	319 (3.48)	75 (4.42)	194 (3.31)	5 (1.26)
Deep Incisional surgical site infection	134 (0.78)	69 (0.75)	12 (0.71)	52 (0.89)	1 (0.25)
Organ/space surgical site infection	89 (0.52)	49 (0.53)	9 (0.53)	30 (0.51)	1 (0.25)
Wound disruption	47 (0.27)	26 (0.28)	5 (0.29)	16 (0.27)	0
DVT/thrombophlebitis	22 (0.13)	13 (0.14)	3 (0.18)	6 (0.10)	0
Pulmonary embolism	159 (0.93)	82 (0.89)	14 (0.83)	61 (1.04)	2 (0.50)
Embolia pulmonar	130 (0.76)	72 (0.79)	43 (0.73)	14 (0.83)	1 (0.25)

Results expressed as n or n (%). * Number of patients with complication (percent of total); ^†^ missing data points, percentage of available data. ORIF/Pros: open reduction internal fixation/prosthetic placement. THA: otal hip arthroplasty; HA: hemiarthroplasty; CVA: cerebrovascular accident; DVT: deep vein thrombosis.

## DISCUSSION

Hip fractures in the geriatric population are common injuries treated by orthopedic surgeons. With aging populations, an absolute increase in the number of hip fractures is anticipated in the coming years. Given that geriatric patients usually have underlying comorbidities, hip fractures in this population are associated with high degrees of morbidity and mortality.^(^ ^3^ ^)^ Treatment options available include non-operative treatment, internal fixation, THA, and HA. The purpose of our study was to analyze the trends in demographics and medical coding used for surgical technique of elderly patients, as well as to compare postoperative complication rates between surgical techniques in this patient population.

Our results suggest that open reduction internal fixation/prosthetic placement was the most commonly logged procedure code for treatment of femoral neck fractures in the elderly, from 2006 to 2015. Hemiarthroplasty ranked second and THA, third. Cannulated screw fixation was least common, and was not coded after the year 2011. The total number of operatively treated neck fractures in geriatric patients increased between 2006 and 2015, with open reduction internal fixation/prosthetic placement code accounting for an increasing fraction of cases each year. Additionally, we found some differences among the surgical techniques as to the probability of developing postoperative pneumonia, requiring postoperative blood transfusion and reoperation. The lowest probability was observed in patients treated with cannulated screw fixation. We believe that this finding is largely attributed to the fact that many surgeons rely on this form of fixation for healthier patients, who present with less complicated fractures.

Several studies have looked at the trends in type of surgery performed for fixation of femoral neck fractures. Miller et al. studied the Medicare database from 1991 to 2008, and found that HA was the most common treatment modality for femoral neck fractures, with 63.9% of patients treated from 2006 to 2008.^(^ ^16^ ^)^ Similarly, studies of the Nationwide Inpatient Sample (NIS) database, from 1990 to 2010, reported that HA was the most common surgical technique across all age groups, accounting for 62.3 to 75.3% of femoral neck fracture surgeries.^(^ ^15^ ^,^ ^17^ ^)^ Our study also found that HA was more common than THA or internal fixation, but only 34.2% of our patients underwent HA. Instead, open reduction internal fixation/prosthetic placement was the most common procedure type among our patients. Miller et al., reported a steady decline in overall procedure volume from 1996 to 2008, but no change in the percentage of fractures treated with each treatment modality over time.^(^ ^16^ ^)^ In contrast, our study showed a steady overall increase in the number of cases performed each year, from 2006 to 2014. Jain et al. analyzed the NIS database from 1990 to 2001 and reported increased utilization of HA and decreased utilization of internal fixation and THA during this time.^(^ ^15^ ^)^ Bishop et al. reported an overall increase in the rates of THA and HA, and decrease in internal fixation cases, from 1998 to 2010.^(^ ^17^ ^)^ Bishop et al. also reported that THA utilization increased in patients aged 60 to 79 years, and decreased in patients aged 80 years and over. In terms of HA utilization, Bishop et al. found that this technique decreased in patients aged 60 to 69 years and increased in patients aged 80 years and up.^(^ ^17^ ^)^ Similar to Bishop et al.,^(^ ^17^ ^)^ we also found that THA and HA utilization increased, while cannulated screw fixation decreased. To our knowledge, the finding that the percent of cases using open reduction internal fixation/prosthetic placement procedures increased has not been reported in prior studies.

Open reduction internal fixation/prosthetic placement was the most commonly used and fastest growing procedure code from 2006 to 2015, and accounted for over half of all cases in 2015 (2,494 out of 4,170 cases). Since this code is non-specific for surgical technique and probably involves all procedure types, this trend suggests that American surgeons prefer to use simpler coding, which does not require specifying the surgical technique.

On the other hand, we consider that the work relative value units (RVU) are partially responsible for the trends of medical coding utilization. Open reduction internal fixation/prosthetic placement (CPT 27236) has a work RVU of 17.61, with a relative frequency of 53.6%, while HA (CPT 27125) with a work RVU of 16.64 had a frequency of 34.2%, from 2006 to 2015. As we can notice, there is a coding redundancy and, according to that, a HA can be coded with either codes. As open reduction internal fixation/prosthetic placement code has higher reimbursement, it may be leading to the observed relative frequency.

Alternatively, this trend may be a result of inadequate understanding of medical billing. Several studies have proposed the need to implement or improve formal education in billing and coding during orthopedic surgery residency training.^(^ ^18^ ^-^ ^20^ ^)^ Regardless of the cause, continuing trends toward simplified coding could be detrimental to the database research community, since a single code cannot be analyzed to compare outcomes between techniques. Thus, the ease and time efficiency of simplified coding needs to be balanced by the need for useful coding data for future research purposes, and increased efforts to train physicians in coding should be implemented.

Many studies have assessed complication rates in elderly patients treated for femoral neck fractures. Overall, thirty-day complication rates between 13.9% and 36.4%,^(^ ^21^ ^-^ ^24^ ^)^ and reoperation rates between 3.6% and 16%^(^ ^22^ ^,^ ^24^ ^,^ ^25^ ^)^ have been reported in recent literature, which are similar to the rates in our study. While previous studies have reported internal fixation as a less optimal treatment,^(^ ^4^ ^,^ ^5^ ^,^ ^25^ ^)^ patients in our study treated with cannulated screw fixation had the lowest complication rates, including reoperations. However, this may be in part accounted for by missing data and the fact that long-term outcomes are not considered in the ACS NSQIP^®^ database.

This study had some limitations. First, we recognize the limitations associated with all database studies. This study, which utilizes the NSQIP^®^ database, is additionally limited in that it only considers 30-day outcomes. Next, it seems likely that missing data limited this study’s ability to adequately assess some variables between groups, especially in respect to cannulated screw fixation operations. Another major limitation involves the CPT coding involved in this study. First, we recognize that the open reduction internal fixation/prosthetic placement group likely contains patients treated with all three surgical techniques. We also speculate that the code for cannulated screw fixation was discontinued in favor of the open reduction internal fixation/prosthetic placement code after 2011, which would account for the fact that the number of patients dropped abruptly to zero in a single year. It is unclear how much this transition may have affected our results. We were also limited by known and unknown baseline differences between groups. For example, the average body mass index of patients treated with THA was overweight, whereas all other groups had average body mass index within more normal weight limits. Additionally, there may be numerous other confounding demographic variables that were not adequately assessed in our study. Surgeons may have chosen healthier, less complicated patients for cannulated screw fixation, which would explain the lower incidence of certain complications among these patients.

Despite these limitations, this study provides valuable insight into recent surgical trends in the management of femoral neck fractures in the elderly. Hospitals, doctors, researchers, medical billers, and patients can utilize data from this study to compare practice standards, both domestically and internationally. In addition, we hope this study will lead to improvements in medical coding and billing in the future, specifically through improved communication between hospital administrators and surgical trainees. Future endeavors to more optimally align the demands of hospital billing and evidence-based research may be an avenue to improve both patient and physician satisfaction.

This study provides valuable insight, for hospitals, physicians, researchers, medical billers and patients, into recent surgical trends in the management of femoral neck fractures in the elderly.

## CONCLUSION

Demographics and morbidity in neck fractures in the elderly did not change significantly from 2006 to 2015, and open reduction internal fixation was the most common treatment, followed by hemiarthroplasty.
